# Critical Conditions Regulating the Gelation in Macroionic Cluster Solutions

**DOI:** 10.1002/advs.202308902

**Published:** 2024-03-02

**Authors:** Xiaohan Xu, Yuqing Yang, Yifan Zhou, Kexing Xiao, Jennifer E. S. Szymanowski, Ginger E. Sigmon, Peter C. Burns, Tianbo Liu

**Affiliations:** ^1^ School of Polymer Science and Polymer Engineering The University of Akron Akron OH 44325 USA; ^2^ Department of Civil and Environmental Engineering and Earth Sciences University of Notre Dame Notre Dame IN 46556 USA; ^3^ Department of Chemistry and Biochemistry University of Notre Dame Notre Dame IN 46556 USA

**Keywords:** counterion‐mediated attraction, gelation transition, hydrogel, macroion, polyoxometalate

## Abstract

The critical gelation conditions observed in dilute aqueous solutions of multiple nanoscale uranyl peroxide molecular clusters are reported, in the presence of multivalent cations. This gelation is dominantly driven by counterion‐mediated attraction. The gelation areas in the corresponding phase diagrams all appear in similar locations, with a characteristic triangle shape outlining three critical boundary conditions, corresponding to the critical cluster concentration, cation/cluster ratio, and the degree of counterion association with increasing cluster concentration. These interesting phrasal observations reveal general conditions for gelation driven by electrostatic interactions in hydrophilic macroionic solutions.

## Graphical abstract

A triangle‐shaped gel region outlining three critical gelation boundaries is identified in the phase diagram of some heavily charged, nanoscale uranyl peroxide macroions in dilute aqueous solutions. This gelation is dominantly driven by counterion‐mediated attraction among the macroions, which causes the self‐assembly of macroions into 2‐D sheets that are further stacked into the supramolecular hydrogel network.

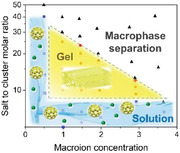

## 1 Introduction

Hydrophilic macroions are 1–6 nm in size and are soluble ions with different solution behaviors from simple ions or colloids. Typical macroions include a variety of charged macromolecules, e.g., polyoxometalate (POM) molecular clusters, polyhedral oligomeric silsesquioxane, metal‐organic cages, dendrimers, functionalized fullerenes, cyclodextrins, organic‐inorganic hybrids, etc.^[^ ^1^, ^2^, ^3^, ^4^, ^5^ ^]^ These macroionic species sharing similar solution behaviors have been developed into materials to solve modern concerns in fields of energy,^[^ ^6^, ^7^ ^]^ environment,^[^ ^8^, ^9^ ^]^ and biology.^[^ ^10^ ^]^

**Figure 1:**
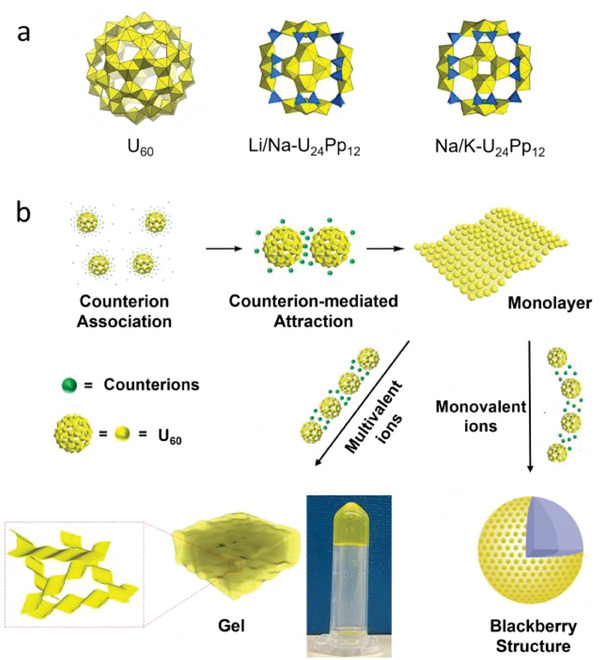
a) Polyhedral representations of uranyl peroxide cage clusters: 2.5‐nm‐size U_60_ (Li_68_K_12_(OH)_20_[UO_2_(O_2_)(OH)]_60_), 1.8‐nm‐size Li/Na‐U_24_Pp_12_ (Li_36_Na_12_[(UO_2_)_24_(O_2_)_24_(P_2_O_7_)_12_]), and 1.8‐nm‐size Na/K‐U_24_Pp_12_ (Na_43_K_8_[(UO_2_)_24_(O_2_)_24_(P_2_O_7_)_12_]). Uranyl polyhedra are shown in yellow. Blue polyhedra represent [P_2_O_7_]^4−^and [HPO_3_]^2−^. b) Schematic illustration showing the mechanism of self‐assembly of uranyl clusters with mono‐, di‐ and trivalent counterions into “blackberry” solutions or hydrogels.^[^ ^13^ ^]^

In dilute aqueous solutions, the size disparity between macroions and their counterions leads to moderate counterion association around macroions^[^ ^11^, ^12^ ^]^ and, consequently, tunable counterion‐mediated attraction, as confirmed by experiments and theoretical calculations.^[^ ^13^, ^14^, ^15^, ^16^, ^17^ ^]^ This force brings like‐charged macroions together, while van der Waals forces,^[^ ^18^, ^19^ ^]^ hydrogen bonding,^[^ ^20^ ^]^ and other forces are proved to be very minor. The counterion‐mediated attraction leads to the formation of 2‐D sheets in solution.^[^ ^21^, ^22^ ^]^ For example, for {U_60_}^60−^ as shown in **Figure** **1**a,b,^[^ ^23^ ^]^ if monovalent counterions are used, the 2‐D sheets will eventually bend and be enclosed to form hollow, spherical blackberry‐type self‐assembled structures, which are commonly observed in many macroionic systems. However, with stronger counterions (di‐ or tri‐valent), the sheets are tough to bend and would remain as standalone sheets in solutions. Their large size and anisotropic nature lead to gelation (similar to graphene gels). These gels are different from conventional colloidal gels, supramolecular gels formed by low molecular weight gelators, or polyelectrolyte gels, in terms of the type and feature of gelators, the driving force, and the building blocks for the network. Colloidal gels typically form when colloidal particles form clusters with a fractal structure manifested in branched and interconnected networks under short‐ranged but strong attractions, such as van der Waals forces and depletion interactions.^[^ ^24^, ^25^, ^26^ ^]^ Most supramolecular gels are constructed by the mutual entanglement and cross‐linking of 1‐D fibers assembled from low molecular weight gelators driven by short‐range interactions, e.g., hydrogen bonding, hydrophobic interactions, π‐π interactions, etc.^[^ ^27^, ^28^ ^]^ Polyelectrolyte gels are charged polymer networks with charges fixed on the polymer chains and small counterions localized in the network.^[^ ^29^, ^30^ ^]^ Although the effect of salts and polyelectrolytes on gels containing polyelectrolytes has been extensively studied,^[^ ^31^, ^32^, ^33^, ^34^ ^]^ different structures and properties of hydrophilic macroions endow them with entirely different responses to additional salts.

In this work, we use several uranyl peroxide clusters as models to demonstrate the critical transition conditions to the gel phase in macroionic solutions. These macroions, with well‐characterized structures, exhibit fascinating physical and chemical properties.^[^ ^35^, ^36^, ^37^, ^38^, ^39^, ^40^, ^41^ ^]^ Here, {U_24_Pp_12_}^48−^ clusters, [(UO_2_)_24_(O_2_)_24_(P_2_O_7_)_12_]^48−^,(Figure 1a) with Y^3+^ counterions, are used as an example to show the detailed studies.

## 2 Results and Discussion

**Figure 2:**
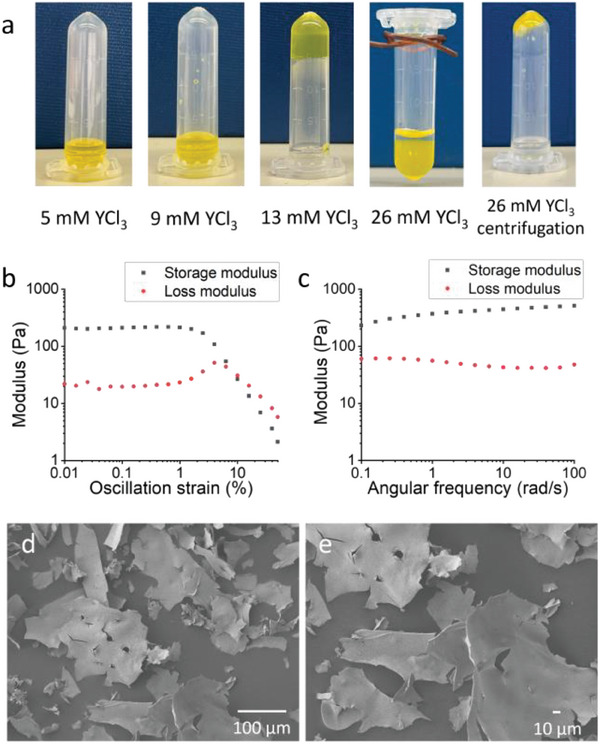
a) Tube inversion tests of a 1.5 mm Li/Na‐U_24_Pp_12_ solution with different amounts of YCl_3_; b,c) Rheological tests of a gel with 1.5 mm Li/Na‐U_24_Pp_12_ and 15 mm YCl_3_; d,e) SEM images of a gel with 2 mm Li/Na‐U_24_Pp_12_ and 15 mm YCl_3_.

**Figure 3:**
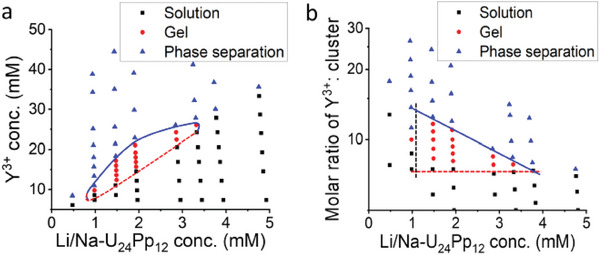
Li/Na‐U_24_Pp_12_ clusters‐YCl_3_ binary phase diagrams with a) the concentration of Y^3+^ and b) the molar ratio of Y^3+^ to clusters as ordinate, respectively.

The aqueous solution of Li/Na‐U_24_Pp_12_ clusters is yellow and transparent over a considerable concentration range due to the good solubility of the cluster. The yellow solution evolves into a transparent yellow gel phase after introducing 13 mM YCl_3_ into a 1.5 mm (≈15 mg mL^−1^) Li/Na‐U_24_Pp_12_ solution (**Figure** **2**a; Figure S1, Supporting Information). Gelation occurs in minutes, and the gel grows stronger and stronger hours after adding salts. Rheological experiments show a storage modulus, G′ ≈ 210 Pa, and a loss modulus, G″ ≈ 20 Pa, much smaller than G′ (Figure 2b). G′ exceeds G″ by an order of magnitude, with both G′ and G″ invariant with frequency (Figure 2c), suggesting the typical feature of a hydrogel. The continuous addition of YCl_3_ gives rise to opaque yellow gels. Moreover, at YCl_3_ concentrations >20 mm, a macrophase separation (syneresis) occurs (Figure 2a and Figure S2, Supporting Information), presumably due to the high crosslinker (Y^3+^) concentration (excess of crosslinker agent) in the gel system.^[^ ^42^ ^]^ The rheological test shows that the bottom phase demonstrates weak gel‐like behavior at high frequencies (Figure S3, Supporting Information). Under centrifugation, the gel feature of the bottom phase is enhanced, forming a stronger gel sticking on the tube bottom. (Figure 2a) Li/Na‐U_24_Pp_12_ solutions experience a similar phase‐transition process (sol to gel to phase separation) upon adding Y^3+^, compared with adding Y^3+^ to U_60_ solutions.^[^ ^23^ ^]^ The possibilities that Van der Waals interaction^[^ ^18^, ^19^ ^]^ or hydrogen bonding^[^ ^20^ ^]^ plays the determining role in the self‐assembly of similar macroions in solutions have been excluded by our early works; thus, we expect the same gelation mechanism as in U_60_ solutions, i.e. the gelation is dominantly driven by counterion‐mediated attraction.^[^ ^23^ ^]^ Scanning electron microscopy (SEM) images of freeze‐dried samples show the existence of small nanosheets in a solution containing 2 mm Li/Na‐U_24_Pp_12_ and 5 mm YCl_3_ (Figure S4) and large nanosheets in a transparent gel with 2 mm Li/Na‐U_24_Pp_12_ and 15 mm YCl_3_ (Figure 2d,e). The sheets in gels sometimes extend to larger than 200 µm, and the curvature of the sheets suggests that they are soft and flexible. By titrating concentrated YCl_3_ solution into Li/Na‐U_24_Pp_12_ cluster solutions at different concentrations, a phase diagram was constructed, showing regions of solution, gel, and macrophase separation. As in **Figure** **3**a, all the phase transitions depend on the concentrations of clusters and Y^3+^ ions, showing a gel area shape akin to that for U_60_/Y^3+^ solutions. When adding divalent salt to Li/Na‐U_24_Pp_12_ solutions, SrCl_2_ cannot trigger gelation and leads to phase separation or precipitation directly, while BaCl_2_ can only help {U_24_Pp_12_} clusters form very weak gels in a narrow concentration range of clusters and salts. We attribute the weakness of divalent salts to their weaker electrostatic attraction to macroions as there is no difference in any other interaction. All these phenomena reveal that the gelation mechanism of Li/Na‐U_24_Pp_12_ with YCl_3_ follows the nanosheet packing model driven by counterion‐mediated attraction as recently reported for the U_60_/Y^3+^ system.^[^ ^23^ ^]^

When Na^+^ or K^+^ are the original counterions, the aqueous solutions of {U_24_Pp_12_} go through an overall similar phase transition process upon adding Y^3+^ compared with Li/Na‐U_24_Pp_12_ (Figure S5, Supporting Information) but with different macroscopic performances. Gels from Na/K‐U_24_Pp_12_ exhibit much lower stiffness and stability than gels from Li/Na‐U_24_Pp_12_. For instance, the rheological test shows the storage modulus and loss modulus of a gel containing 1 mm Na/K‐U_24_Pp_12_ and 13 mm YCl_3_ are ≈50 and ≈5 Pa, respectively (Figure S6, Supporting Information).

It is expected that the cluster solution with a higher concentration needs more YCl_3_ for gelation and phase separation (Figure 3a). When applying the molar ratio of Y^3+^ to clusters as the Y‐axis, a triangle‐shaped gel region appears, enabling us to define the critical boundaries for gelation in the macroion solutions. (Figure 3b) The gel region exhibits three boundaries on the phase diagram regarding this system.

First, the critical gelation concentration for Li/Na‐U_24_Pp_12_ cluster is about 1.0–1.5 mm (10–15 mg mL; 1–1.5 wt.%), which is independent of the amount of Y^3+^ present in solution (Figure 3b, black dashed line). This represents the minimum amount of macroions needed for gelation.

Second, at different cluster concentrations, there exists a critical gelation concentration for Y^3+^ (Figure 3a, red dashed line), which is fixed around the molar ratio of Y^3+^/Li/Na‐U_24_Pp_12_ at 8, equivalent to a total relative charge ratio of 1:2 (Figure 3b, red dashed line). That is, ≈50% of multivalent counterions in total charge number is required for gelation, which is independent of macroionic concentration. Thus, we conclude that forming a gel network always requires a certain amount of multivalent counterions. These counterions not only attract macroions together to form 2‐D nanosheets but also play a role in organizing and packing these nanosheets into the homogeneous gel network.

Third, the Y^3+^ concentration required to transition from gel to macrophase separation increases with increasing cluster concentration (Figure 3a, blue curve). Whereas, if examined by the molar ratio of Y^3+^/Li/Na‐U_24_Pp_12_, the transition molar ratio decreases with increasing cluster concentration. Consequently, two boundaries (the red and the blue line in Figure 3b) will be linked to form an enclosed area that regulates the gelation region. Therefore, in this Y^3+^/Li/Na‐U_24_Pp_12_ system, gelation only occurs over a range of cluster concentrations, and a lower Y^3+^: U_24_Pp_12_ molar ratio is needed for phase separation at higher cluster concentrations. This decreasing tendency is probably due to the increasing degree of congestion in the system, which enables more and more sheets to share their counterions and further stack.

**Figure 4:**
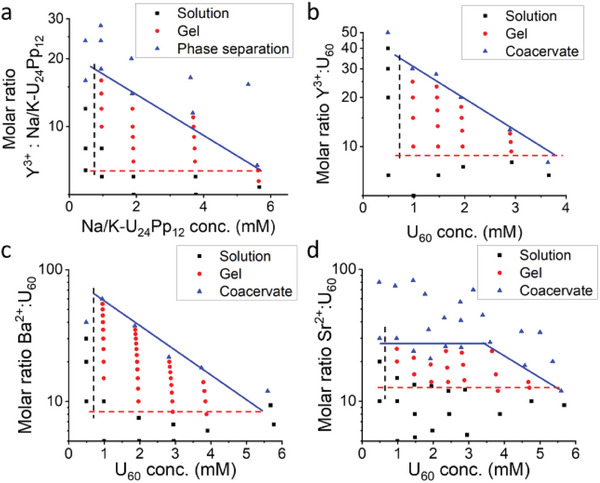
Binary phase diagrams with molar ratio of multivalent counterion to cluster as ordinate: a) Na/K‐U_24_Pp_12_ clusters and YCl_3_; b) U_60_ clusters and YCl_3_; c) U_60_ clusters and BaCl_2_; d) U_60_ clusters and SrCl_2_.

Similar‐shaped gel regions appear in other macroionic solutions. These macroionic solutions demonstrate a spindle‐shaped gel region when applying Y^3+^ concentration as the Y‐axis (Figure S7, Supporting Information) and a triangle‐shaped gel region when using the Y^3+^/cluster molar ratio as the Y‐axis. (**Figure** **4**) This macroscopic resemblance arises from the same mechanism of gelation in these solutions. Moreover, differences among macroions or counterions lead to different boundary positions in the phase diagrams.

We first compare the gelation involving Y^3+^. The minimum cluster concentrations of Na/K‐U_24_Pp_12_ and U_60_ required for gelation are also about 1 mm, which is comparable to that in the case of Li/Na‐U_24_Pp_12_/Y^3+^ solution (1.0–1.5 mm), showing that the critical cluster concentration for the gelation does not correlate strongly with the type of cluster. When comparing the critical gelation molar ratio, each Na/K‐U_24_Pp_12_ cluster needs 6–7 Y^3+^ to form a gel network, while each U_60_ cluster needs ≈10 Y^3+^ (red dashed lines in Figure 4a,b). Thus, the total charge ratio of Y^3+^/Na/K‐U_24_Pp_12_ on the boundary is about 20:48 with ≈42% of charges balanced on the cluster, and the total charge ratio of Y^3+^/U_60_ is about 30:60 with ≈50% of charges balanced on the cluster, also similar to the scenario of Li/Na‐U_24_Pp_12_/Y^3+^ gels. Furthermore, Na/K‐U_24_Pp_12_/Y^3+^ and U_60_/ Y^3+^ gels at higher cluster concentrations also collapse and phase separate at a smaller molar ratio of salt to clusters. These gel‐to‐bi‐phase transition points were fitted into a linear regression to compare the impact of additional counterions on the gel (blue lines in Figure 3b, Figure 4a,b). The slope of the linear regression in Li/Na‐U_24_Pp_12_/Y^3+^, Na/K‐U_24_Pp_12_/Y^3+^, and U_60_/Y^3+^ systems is −2.0, −2.3, and −11.5 respectively. The two {U_24_Pp_12_} clusters have comparable slopes, indicating the comparable response of their gels to the addition of Y^3+^ due to their similar cluster structures. On the contrary, the U_60_/Y^3+^ system might derive a much steeper slope from its heavier charges than the other two clusters, which endows U_60_ clusters with stronger affinity to Y^3+^.

These critical gelation boundaries are not exclusive for Y^3+^ counterions; instead, we also identified similar boundaries in U_60_ and Ba^2+^ or Sr^2+^ systems. As in Figure 4c,d, the critical gelation concentration of U_60_ is ≈1 mm regardless of the type of counterion. The critical gelation molar ratio is ≈9 for Ba^2+^ and ≈12 for Sr^2+^, which suggests a stronger affinity of Ba^2+^ than Sr^2+^. Especially, the transition from gel to coacervate in U_60_/Sr^2+^ systems demonstrated a flat part and then decay as in other systems. We speculate that the weaker interaction between Sr^2+^ and U_60_ causes the assembled nanosheets to be smaller and less extensible, thus making it difficult to share additional Sr^2+^ and eventually leading to the flat part.

The three gelation boundaries in macroionic solutions show features similar to the gelation with two‐component‐mixed cross‐link junctions proposed by Tanaka: for a ternary solution with two types of multifunctional solute with gelation capability, their critical sol‐gel transitions are independent of the relative concentrations of the two species or the volume fraction of the primary species (the one with more functional groups).^[^ ^43^ ^]^ Our work implies that counterion‐mediated interaction could turn these nanoscale inorganic macroions into sturdy building blocks for constructing gel networks, like other well‐explored multifunctional gelators with capabilities for hydrogen bonding, π‐π stacking, etc. It is interesting that these gels based on electrostatic interaction‐driven nanosheets exhibit similar critical sol‐gel transition boundaries as the gels formed from simple “cross‐linking” of two multifunctional solutes.^[^ ^43^ ^]^ Electrostatic interactions usually serve as driving forces for gelation together with other non‐covalent forces,^[^ ^44^, ^45^, ^46^ ^]^ except for a recent discovery of a hydrogel based on 1‐D fiber‐like branched structures from inorganic macroions {Mo_7_O_24_}^6−^ and Fe^3+^ through counterion‐mediated interaction.^[^ ^47^ ^]^ Although electrostatic interactions are strong in polyelectrolyte gels, for low molecular weight electrolytes, electrostatic interactions are usually weaker and result in ionic networks acting like ionic liquids.^[^ ^48^, ^49^ ^]^ To our knowledge, the hydrogels we report here are rare gel examples from small inorganic gelators via a hierarchical construction through 2‐D nanosheets by electrostatic interactions. Theories of phase transitions in polymer gels^[^ ^50^, ^51^, ^52^, ^53^ ^]^ have well‐described their thermodynamics, kinetics, collapse, etc., while theories in colloidal gels paid more attention to gelation routes and driving forces with colloid state diagrams regarding different phases.^[^ ^24^, ^26^, ^54^ ^]^ With this work, we hope to provide a fundamental understanding of this new type of physical gel preliminarily by outlining their critical gelation boundaries.

## 3 Conclusion

In summary, we identified gelation in dilute aqueous solutions of {U_24_Pp_12_} clusters based on the packing of 2‐D nanosheets driven by counterion‐mediated interaction. This newly discovered gelation behavior has also been observed in solutions of different macroions and salts, which could be a universal feature for heavily charged macroions. We further explored the critical conditions regulating the gelation in macroionic cluster solutions with different POM clusters and salts. There are three critical boundaries regarding the gel region: i) the minimum concentration of macroions required for gelation is about 1–1.5 mm and is independent of counterion concentration; ii) the critical molar ratio of macroion/counterion on the sol‐gel transition boundaries depends on types of macroion and salt and remain constant for a specific combination; iii) the critical molar ratio of macroion/counterion on the transition from gel to macrophase separation decreases as macroion concentration increasing, thus closing the gel region and forming a triangular gel region. We investigated five combinations of macroion and salt, including three types of POM clusters and three types of salts, finding that all share the same phase transition features. We believe these critical boundaries are a common feature for these novel hydrogels generated from macroions and simple salts and will open a new door in developing supramolecular nanomaterials.

## 4 Experimental Section

See Supporting Information.

## Conflict of Interest

The authors declare no conflict of interest.

## Supporting information

Supporting Information

## Data Availability

The data that support the findings of this study are available from the corresponding author upon reasonable request.
