# Supplementary material for: Critical Conditions Regulating the Gelation in Macroionic Cluster Solutions
Source: Adv Sci (Weinh). 2024 Mar 2;11(18):2308902. doi: 10.1002/advs.202308902 (PMC11095157; doi:10.1002/advs.202308902)
Supplement: Supplementary file 1 — Supporting Information [file ADVS-11-2308902-s001.pdf]

ADVANCED SCIENCE
Open Access

Supporting Information

for *Adv. Sci.*, DOI 10.1002/advs.202308902

Critical Conditions Regulating the Gelation in Macroionic Cluster Solutions

Xiaohan Xu, Yuqing Yang, Yifan Zhou, Kexing Xiao, Jennifer E. S. Szymanowski, Ginger E. Sigmon, Peter C. Burns and Tianbo Liu**

Supporting information for

Critical Conditions Regulating the Gelation in Macroionic Cluster Solutions

Xiaohan Xu,[a] Yuqing Yang,[a] Yifan Zhou,[a] Kexing Xiao,[a] Jennifer E.S. Szymanowski,[b] Ginger E. Sigmon,[b] Peter C. Burns,*[b,c] Tianbo Liu* [a]

[a] X. Xu, Dr. Y. Yang, Y. Zhou, K. Xiao, Prof. T. Liu
School of Polymer Science and Polymer Engineering
The University of Akron
Akron, OH, 44325 USA
E-mail: tliu@uakron.edu

[b] J. E.S. Szymanowski, Dr. G. E. Sigmon, Prof. P. C. Burns
Department of Civil and Environmental Engineering and Earth Sciences
University of Notre Dame
Notre Dame, Indiana 46556, United States
E-mail: pburns@nd.edu

[c] Prof. P. C. Burns
Department of Chemistry and Biochemistry
University of Notre Dame
Notre Dame, Indiana 46556, United States

Table of Contents

1. Chemicals, instruments, and experimental methods
2. Supplemental data
3. References

1. Chemicals, instruments, and experimental methods

<u>Chemicals</u>

Polyoxometalate clusters were synthesized according to the procedure in the literature.[1,2] All the salts were purchased from Sigma-Aldrich and used without purification. Deionized (DI) water was used to prepare the samples.

<u>Instruments</u>

ARES-G2 Rheometer from TA instrument. The rheological tests shown in Figure 2 were performed using cone and plate geometry (25 mm 0.04 rad), and others were performed using 25 mm parallel plate geometry. For strain sweep, the angular frequency was fixed at 10 rad/s. For frequency sweep, the strain was fixed at 1% or 0.5%.

Scanning Electron Microscopy (SEM). SEM images were taken from a JEOL JCM-6000PLUS NeoScope Benchtop SEM using a secondary electron detector (SED) with an accelerating voltage of 5 to 10 kV. Samples were freeze-dried before imagining them.

<u>Experimental methods</u>

The gel tests and sample preparation followed the published procedure.[3] Crystal samples of U_{60} and $\{U_{24}Pp_{12}\}$ clusters were dissolved in DI water to prepare concentrated stock solutions which were diluted into appropriate concentrations for gel tests. All salts (YCl_3, $BaCl_2$, and $SrCl_2$) were dissolved into solutions with concentrations of 0.5 and 1 mM. After each addition of salt, the sample tube containing cluster solution was put on a tough mixer for 10 seconds and sonicated for 10 minutes. Then, inversion tests were conducted after 10 minutes of standing. Upon adding YCl_3 to $\{U_{24}Pp_{12}\}$ cluster solution, a small amount of power-like precipitate was observed with the solution maintaining yellow. We attribute the precipitate to the ultra-high local concentration of Y^{3+} right after adding salt. The precipitate can be separated out through centrifugation, and the upper clear yellow solution still shows gelation behavior.

2. Supplemental data

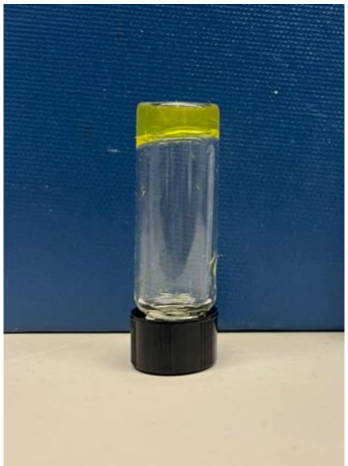

Figure S1. Inversion test of a gel with 1.5 mM Li/Na{$U_{24}Pp_{12}$} and 18 mM YCl_3.

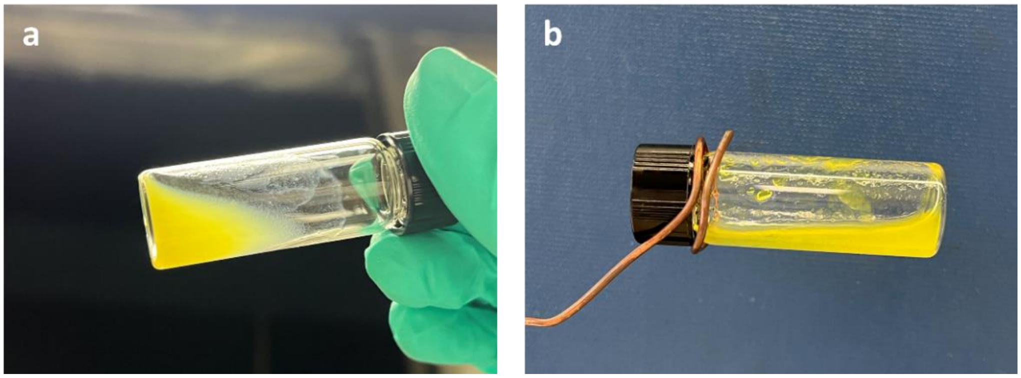

Figure S2. Phase separated systems containing 1.5 mM Li/Na-$U_{24}Pp_{12}$ and 25 mM YCl_3. For (a), the gel test was conducted in plastic vial then transferred to glass vial. For (b), the gel test was directly conducted in glass vial. Although it shows part of components stick on the glass wall during the mixing, the properties of the gel do not change from different vials.

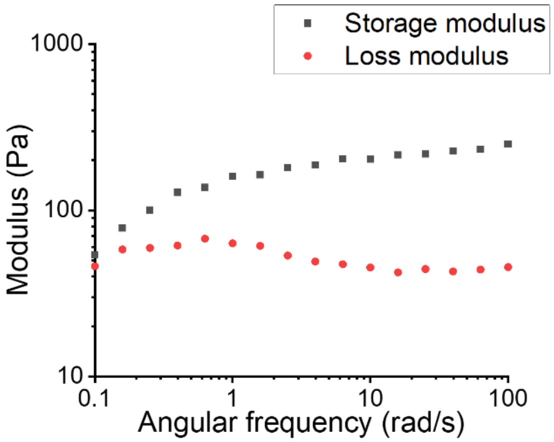

Figure S3. Rheological test of bottom phase with 1.5 mM Li/Na-$U_{24}Pp_{12}$ and 20 mM YCl_3.

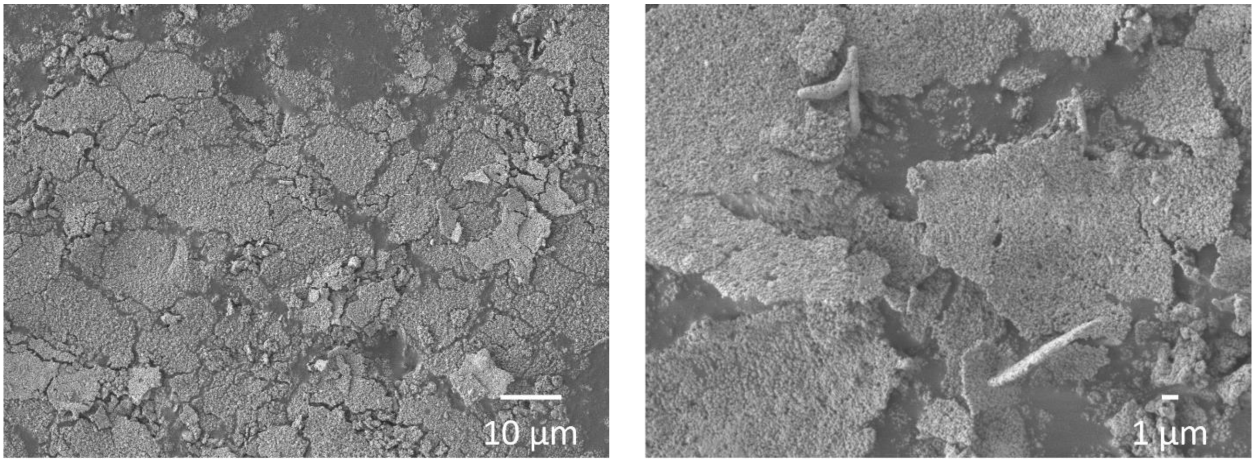

Figure S4. SEM images of a freeze-dried solution sample containing 2.0 mM Li/Na-$U_{24}Pp_{12}$ and 5 mM YCl_3.

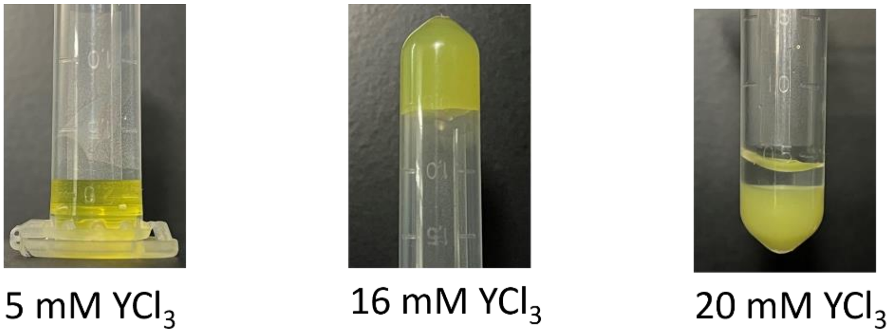

Figure S5. Inversion tests of 2 mM Na/K-$U_{24}Pp_{12}$ with different amount of YCl_3.

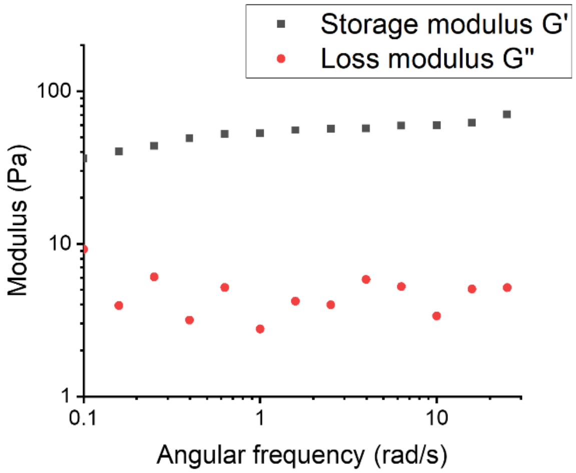

Figure S6. Rheological test of 1 mM Na/K-$U_{24}Pp_{12}$ with 13 mM YCl_3

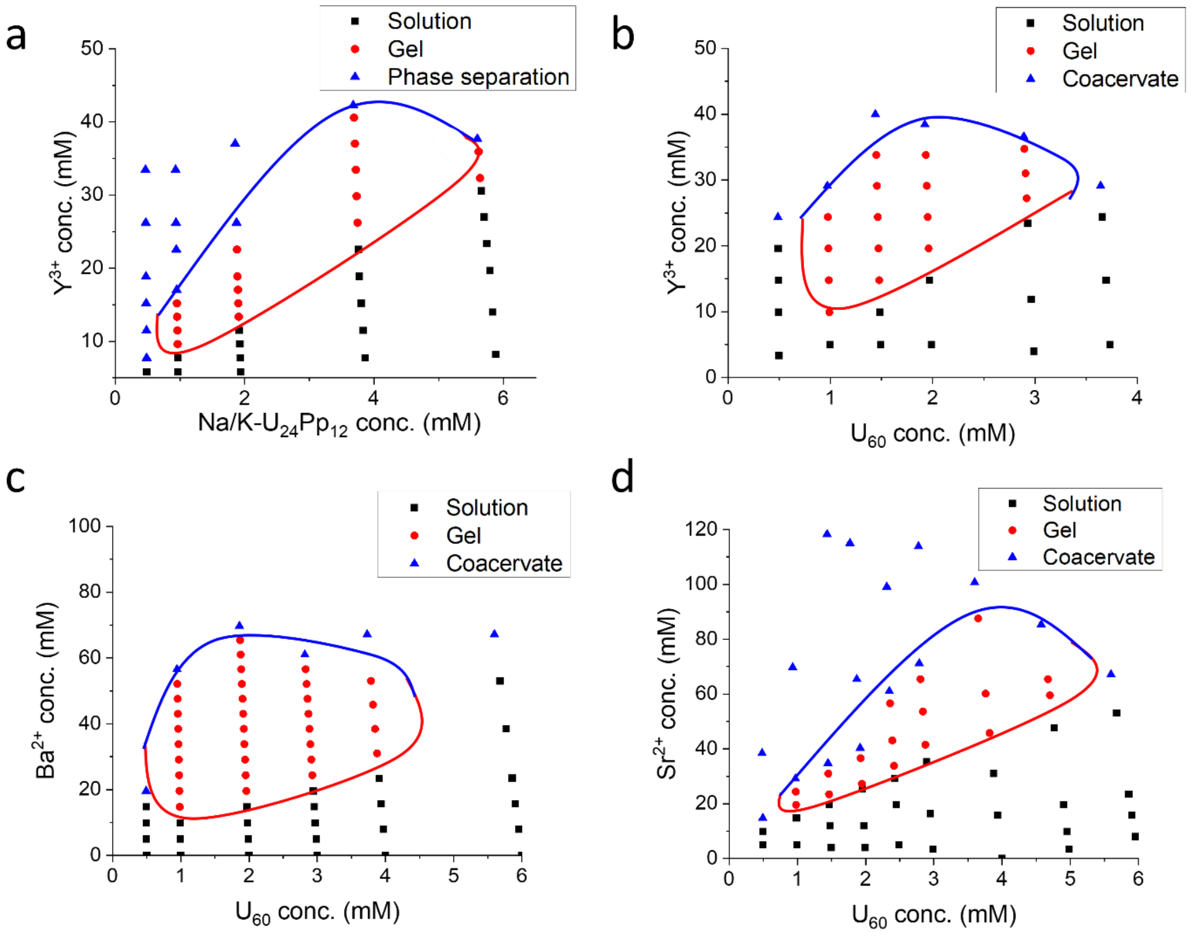

Figure S7. Binary phase diagrams with the concentration of multivalent counterion as ordinate: (a) Na/K-$U_{24}Pp_{12}$ clusters and YCl_3; (b) U_{60} clusters and YCl_3; (c) U_{60} clusters and $BaCl_2$; (d) U_{60} clusters and $SrCl_2$.

3. References

[1] M. Dembowski, T. A. Olds, K. L. Pellegrini, C. Hoffmann, X. Wang, S. Hickam, J. He, A. G. Oliver, P. C. Burns, *J. Am. Chem. Soc.* **2016**, *138*, 8547–8553.

[2] G. E. Sigmon, D. K. Unruh, J. Ling, B. Weaver, M. Ward, L. Pressprich, A. Simonetti, P. C. Burns, *Angew. Chemie Int. Ed.* **2009**, *48*, 2737–2740.

[3] Y. Yang, Y. Zhou, J. Chen, T. Kohlgruber, T. Smith, B. Zheng, J. E. S. Szymanowski, P. C. Burns, T. Liu, *J. Phys. Chem. B* **2021**, *125*, 12392–12397.
